# Functional Ramifications for the Loss of P-Selectin Expression on Hematopoietic and Leukemic Stem Cells

**DOI:** 10.1371/journal.pone.0026246

**Published:** 2011-10-24

**Authors:** Con Sullivan, Yaoyu Chen, Yi Shan, Yiguo Hu, Cong Peng, Haojian Zhang, Linghong Kong, Shaoguang Li

**Affiliations:** 1 Maine Institute for Human Genetics and Health, Bangor, Maine, United States of America; 2 Division of Hematology/Oncology, Department of Medicine, University of Massachusetts Medical School, Worcester, Massachusetts, United States of America; 3 The Jackson Laboratory, Bar Harbor, Maine, United States of America; 4 Dana Farber Cancer Institute, Boston, Massachusetts, United States of America; Emory University, United States of America

## Abstract

Hematopoiesis is a tightly regulated biological process that relies upon complicated interactions between blood cells and their microenvironment to preserve the homeostatic balance of long-term hematopoietic stem cells (LT-HSCs), short-term HSCs (ST-HSCs), multipotent progenitors (MPPs), and differentiated cells. Adhesion molecules like P-selectin (encoded by the *Selp* gene) are essential to hematopoiesis, and their dysregulation has been linked to leukemogenesis. Like HSCs, leukemic stem cells (LSCs) depend upon their microenvironments for survival and propagation. P-selectin plays a crucial role in Philadelphia chromosome -positive (Ph^+^) chronic myeloid leukemia (CML). In this paper, we show that cells deficient in P-selectin expression can repopulate the marrow more efficiently than wild type controls. This results from an increase in HSC self-renewal rather than alternative possibilities like increased homing velocity or cell cycle defects. We also show that P-selectin expression on LT-HSCs, but not ST-HSCs and MPPs, increases with aging. In the absence of P-selectin expression, mice at 6 months of age possess increased levels of short-term HSCs and multipotent progenitors. By 11 months of age, there is a shift towards increased levels of long-term HSCs. Recipients of BCR-ABL-transduced bone marrow cells from P-selectin-deficient donors develop a more aggressive CML, with increased percentages of LSCs and progenitors. Taken together, our data reveal that P-selectin expression on HSCs and LSCs has important functional ramifications for both hematopoiesis and leukemogenesis, which is most likely attributable to an intrinsic effect on stem cell self-renewal.

## Introduction

Hematopoietic stem cells (HSCs) rely upon exquisite interactions with each other and their microenvironments to fulfill their specific cellular functions. HSCs are responsible for the maintenance of the blood cell pool and can fulfill this function because of their unique capacities to self-renew and to differentiate into functioning blood cells. Proper hematopoietic homeostasis is essential in order to preserve the appropriate balance of long-term HSCs (LT-HSCs), short-term HSCs (ST-HSCs), and multi-potent progenitors (MPPs). To achieve this, HSCs must be regulated and maintained through assorted growth factors, cell cycle regulation, transcription factors, epigenetic modulation, and interactions with the microenvironment [1], [2].

P-selectin is a well-studied adhesion protein known to be expressed on the surface of activated endothelial cells and platelets [3]. We have previously shown that P-selectin plays a crucial role in Philadelphia chromosome -positive (Ph^+^) chronic myeloid leukemia (CML) [4]. The mechanism by which the lack of P-selectin was thought to promote the rate of CML appeared to involve the capacity of these leukemic cells to be retained in the bone marrow through adhesive interactions with the stroma. This defect had been thought to be related to the stroma and not to the leukemic stem cell (LSC) itself. Here, we investigate the role of P-selectin plays on HSC- and LSC-containing lin low/Sca-1^+^/c-kit^+^ (LSK) cells. We demonstrate that the loss of P-selectin expression leads to an increased capacity to repopulate the bone marrow in both the short- and long-term. We show that this is likely due to an enhanced capacity for self-renewal. We also demonstrate with our retroviral transduction/transplantation BCR-ABL-induced CML mouse model that the loss of P-selectin expression on LSC-containing LSK cells significantly increased the severity of the disease. We show that aging mice exhibit elevated levels of P-selectin on the surfaces of their LT-HSCs (CD34^−^ LSK) but not their ST-HSCs and MPPs (CD34^+^ LSK).

## Materials and Methods

### Mouse strains

Wild type C57BL/6J mice (WT hereafter), transgenic WT mice ubiquitously expressing eGFP (C57BL/6-Tg[UBC-GFP]^30Scha/J^; UBC-GFP hereafter), congenic WT mice expressing CD45.1 (B6.SJL-*Ptprc^a^ Pepc^b^*/^BoyJ^; CD45.1 hereafter), and WT mice with homozygous null mutations in the gene encoding the P-selectin protein (B6.129S7-Selp^tm1Bay^; *Selp* ^−*/*−^ hereafter) were obtained from The Jackson Laboratory (Bar Harbor, ME) and maintained in accordance with the standards set forth by the institution's Animal Care and Use Committee.

### Bone marrow transduction and transplantation

As previously described, the cDNA encoding the p210 isoform of BCR-ABL was subcloned into the retroviral *MSCV-IRES-eGFP* (MIG) in order to make the bicistronic MIG-p210 plasmid construct [4], [5], [6]. Helper-free, ecotropic retroviral stocks of MIG and MIG-p210 were generated by transient transfection of 293T/17 cells with the respective constructs. Viral titers were determined by analyzing the percentage of GFP^+^ cells following infection of NIH3T3 cells with serial dilutions of retroviral stock.

A CML-like myeloproliferative disease can be induced following transplantation of bone marrow cells derived from 5-fluorouracil (5-FU)-pretreated donor mice, pre-stimulated with IL3, IL6, and stem cell factor (SCF), and transduced with retrovirus encoding *BCR-ABL* (MIG-p210) [5]. As previously described, 12-week-old female donor mice (*Selp* ^−*/*−^ or WT) were pretreated with 5-FU (200 mg/kg) via lateral tail vein injection [4], [5], [6]. Four days post-injection, donor mice were sacrificed, and bone marrow cells were harvested. Cells were pre-stimulated with IL3, IL6, and SCF and subjected to two rounds of co-sedimentation/infection with high-titer MIG-p210 retroviral stock. Cells were collected and transplanted into irradiated (2 rounds of 550 cGy) WT mice (2×10^5^ cells/mouse). Twelve-week-old female WT recipient mice were closely monitored for outward signs of CML-like disease. For the assay designed to test the role of aging on leukemogenesis, we used 15-month-old *Selp* ^−*/*−^ and WT male mice for donors and transplanted 2.0×10^5^ cells into each irradiated 3-month-old WT male recipient. Hematopoietic tissues and peripheral blood were collected from sick mice at specific time points in order to characterize the onset and development of disease. For the whole bone marrow repopulation study, we transduced 5-FU pretreated bone marrow cells derived from 12 week old female *Selp* ^−*/*−^ and WT mice and pre-stimulated with IL3, IL6, and SCF. These cells were then subjected to two rounds of retroviral infection with a construct encoding GFP (MIG) and transplanted via lateral tail vein injection into irradiated (2 rounds of 550 cGy) 12 week-old female WT mice (2×10^5^ cells/mouse).

### Bone marrow transduction efficiency assay

As previously described, 12-week-old female donor mice (*Selp* ^−*/*−^or WT) were pre-treated with 5-FU (200 mg/kg) via lateral tail vein injection [4], [5], [6]. Four days post-injection, donor mice were sacrificed, and bone marrow cells were harvested. Cells were pre-stimulated with IL3, IL6, and SCF and subjected to two rounds of co-sedimentation/infection with high-titer MIG-p210 retroviral stock. The virus-transduced bone marrow cells were cultured for 48 h. Then, those bone marrow cells were analyzed to determine the percentage of GFP^+^Lin^-^cKit^+^Sca-1^+^ cells by FACS.

### *In vitro* culture of LSCs

Bone marrow cells were isolated from CML mice and were cultured in vitro in the presence of StemSpan SFEM (Stem Cell Technologies, Vancouver, CA), stem cell factor, insulin-like growth factor-2, thrombopoietin, heparin, and fibroblast growth factor, as described previously [7], [8].

### Cell cycle analysis of HSCs

Cell cycle status was assessed by staining cells with 5 u*M* Hoechst blue for 90 minutes. The percentage of HSCs in S+G2M stage was determined by FACS [9].

### Flow cytometry

The percentages of LT-HSCs, LT-LSCs (both CD34^−^ LSK), ST-HSCs+MPPs, ST-LSCs+MPPs (both CD34^+^ LSK), as well myeloid progenitor cells (non-leukemic and leukemic), were determined by flow cytometry following the isolation of bone marrow cells of mice that had been transplanted with cells derived from *Selp* ^−*/*−^ and WT donors. Animals were sacrificed at 14 d post-transplant (in the case of the MIG-p210 transplant) or 18 d post-transplant (in the case of the MIG repopulation transplant) in order to observe changes in these blood cell populations and the disease developed. Prior to analysis, bone marrow cells were incubated in an NH_4_Cl red blood cell lysis buffer (pH 7.4) to facilitate the removal of erythrocytes. Single cells were analyzed for viability by propidium iodide staining. Viable cells were deemed transduced by MIG or MIG-p210 retrovirus if they expressed GFP. In cells transduced by the MIG-p210, cells expressing GFP were deemed leukemic, as this was an indication that BCR-ABL was simultaneously being expressed from the same bicistronic construct. Viable GFP^+^ cells were stained with Lin, Sca1, c-Kit (CD117), CD34, and CD16/32 in order to determine the percentages of stem cells and progenitors attributable to the donor transplantation of cells. Antibody to P-selectin was purchased from eBioscience (San Diego, CA).

### Bone marrow competition assay

Bone marrow cells were isolated from 18–20-week-old transgenic female UBC-GFP mice and *Selp* ^−*/*−^ mice. Erythrocytes were lysed as previously described. HSCs that are Lin low/Sca1^+^/c-kit^+^ (LSK) were isolated and collected by sterile FACS. LSK cells from UBC-GFP and *Selp* ^−*/*−^ mice were mixed in a 1∶1 ratio. Recipient female WT mice were subjected to two rounds of 550 cGy irradiation and injected with 1×10^4^ total LSK cells (5×10^3^ cells of each). The following day, the same recipient female WT mice were injected with whole bone marrow cells derived from CD45.1 female mice to ensure survival. At 30, 45, 86, and 120 d post-transplantation of UBC-GFP and *Selp* ^−*/*−^ cells, peripheral blood was collected and subjected to flow cytometric analysis. CD45.2^+^ cells were determined to be GFP^+^ or GFP^−^. At 120 d post-transplantation, recipient mice were sacrificed, and bone marrow was analyzed to determine the percentage of CD45.2 GFP^+^ and GFP^−^ cells, and more specifically, HSC-containing LSK and LT-HSC.

### Homing experiment

We compared the ability of *Selp* ^−*/*−^ and WT marrow cells to home to the bone marrow compartment of recipients. Bone marrow cells from UBC-GFP and either *Selp* ^−*/*−^ or WT mice (all CD45.2) were 1∶1 mixed and then injected into CD45.1 recipient mice. The percentages of CD45.2^+^GFP^+^ (UBC-GFP) and CD45.2^+^GFP^−^ (*Selp* ^−*/*−^) cells in the bone marrow of CD45.1 recipient mice were compared after 3 h. For the HSC homing assay, we normalized LSK cells and injected around 3×10^6^ bone marrow cells of either WT or *Selp* ^−*/*−^ into CD45.1 recipient mice. The total number of CD45.2^+^ LSK cells was calculated after 3 h.

### 

Statistical analyses were performed using GraphPad Prism v5.01 software for Windows, (GraphPad Software, San Diego, CA USA).

## Results

### Loss of *Selp* expression supports bone marrow repopulation

We previously showed a role for P-selectin in our mouse model for human CML. In that study, we demonstrated that *Selp* ^−*/*−^ recipients of *Selp* ^−*/*−^ donor cells developed CML more quickly than WT recipients of WT donor cells. In this study, we wanted to better understand the role P-selectin plays on donor cell function, irrespective of the recipient stromal contribution, during normal hematopoiesis and during Ph^+^ leukemogenesis in mice.

**Figure 1:**
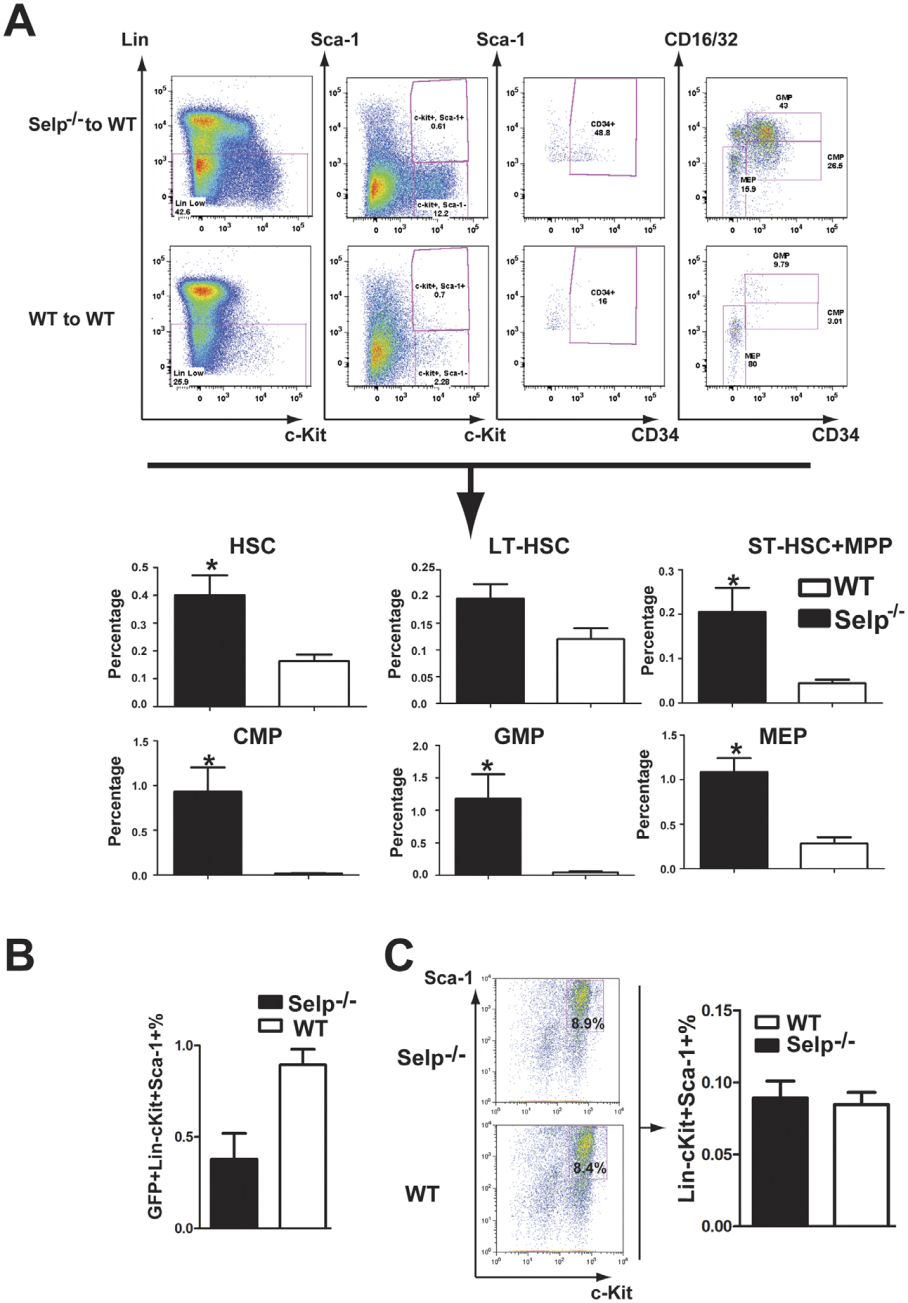
*Selp* ^−*/*−^ donor cells more efficiently repopulate the bone marrow than controls. Representative FACS analyses (top) comparing the bone marrow cell populations of recipients of 5-FU pretreated WT and *Selp* ^−*/*−^ cells pre-stimulated with IL3, IL6, and SCF and transduced twice with a MSCV-based retrovirus expressing GFP (as a marker for evidence of infection) at 18 d post-transplantation. Viable GFP^+^ singlets were stained with antibodies to Lineage (Lin), c-Kit, Sca-1, CD34, and CD16/32. HSCs derived from donors were GFP^+^ and Lin^−^, Sca-1^+^, and c-Kit^+^ (LSK). LT-HSC was GFP^+^, CD34^−^ LSK; and ST-HSC + MPP were GFP^+^, CD34^+^ LSK. CMP were GFP^+^, CD34^+^, Lin^−^, Sca-1^−^, c-Kit^+^, and CD16/32^−^. GMP were GFP^+^, CD34^+^, Lin^−^, Sca-1^−^, c-Kit^+^, and CD16/32^+^, and MEP were GFP^+^, CD34^−^, Lin^−^, Sca-1^−^, c-Kit^+^, and CD16/32^−^. (Bottom) Recipients of *Selp* ^−*/*−^ cells (N = 5) possessed significantly more HSC-containing LSKs than recipients of WT cells (N = 4) (P = 0.0259). There were considerably more LT-HSC cells in the *Selp* ^−*/*−^ recipients, although not statistically significant . Recipients of *Selp* ^−*/*−^ cells had significantly more ST-HSCs + MPPs, CMP, GMP, and MEP . (B) Viral transduction efficiency of WT (WT) and *Selp* ^−*/*−^ HSCs. The bone marrow cell populations of WT and *Selp* ^−*/*−^ were pre-stimulated with IL3, IL6, and SCF, transduced with a MSCV-based retrovirus expressing GFP and cultured for 48 h. Viable GFP+ LSK cells of WT and *Selp* ^−*/*−^ were compared by FACS. (C) The percentage of HSCs between WT and *Selp* ^−*/*−^ after culture for 4 d. The bone marrow cells of WT and *Selp* ^−*/*−^ were cultured under stem cell conditions for 4 d. The percentage of viable LSK cells of WT and *Selp* ^−*/*−^ were compared by FACS. For each experiment, the following denote the statistical significance observed: 0.01<P<0.05 =  *; 0.001<P<0.01 =  **; P<0.001 = ***.

We infected bone marrow cells derived from 5-FU-treated donor WT and *Selp* ^−*/*−^ mice with retrovirus expressing GFP. Irradiated WT recipients were transplanted with 2×10^5^ of these transduced donor cells. At 18 d post-transplant, recipients of *Selp* ^−*/*−^donor cells exhibited significantly higher percentages of HSC-containing LSK cells than the recipients of WT marrow cells. *Selp* ^−*/*−^recipients possessed elevated percentages of LT-HSCs (CD34^−^ LSK) and significantly increased percentages of ST-HSCs + MPPs (CD34^+^ LSK). *Selp* ^−*/*−^ recipients also possessed significantly elevated percentages of CMPs, GMPs, and MEPs (Fig. 1A). These data indicate that the loss of *Selp* expression supports bone marrow cell repopulation in irradiated recipients and thus may impart a functional advantage. These findings were supported by the fact viral transduction efficiencies did not favor the transduction of *Selp* ^−*/*−^cells over WT cells (Fig. 1B) and that the percentages of LSK cells after four days of stem cell culture were very similar (Fig. 1C).

### Loss of *Selp* expression supports HSC long term engraftment

**Figure 2:**
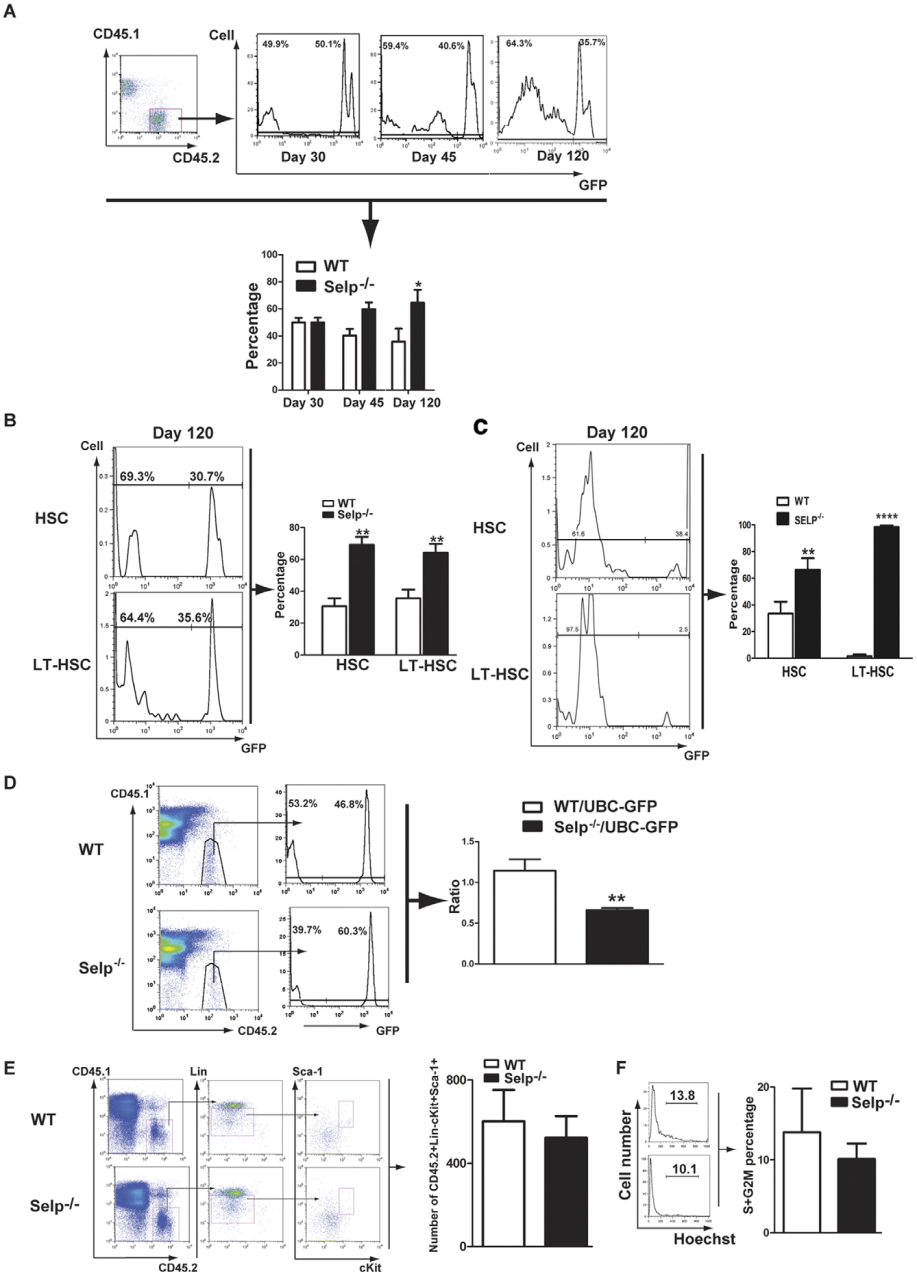
*Selp* ^−*/*−^ donor cells exhibit diminished homing velocities but longer-term repopulation advantages over controls. (A,B) CD45.2 LSK cells derived from *Selp* ^−*/*−^ and UBC-GFP mice were sorted by sterile technique and mixed in a 1∶1 ratio. (A) Peripheral blood was extracted from recipients at 30 d (N = 6), 45 d (N = 6), 86 d (N = 5), and 120 d (N = 3) post-transplantation and subjected to FACS analysis (top). The CD45.2 cell population was assayed, and the percentage of GFP^+^ (UBC-GFP) and GFP- (*Selp* ^−*/*−^) cells were determined. Data are represented as the mean ± SEM. One sample t-tests (theoretical means  = 50%, assuming no competitive advantage) were applied to the data. Statistically significant differences were not observed at 30 d (P = 0.9639) but became apparent at 45 d, 86 d, and 120 d (P = 0.0113). (B) The percentage of HSC-containing LSK cells, and specifically CD34^−^ LSK LT-HSCs, either GFP^+^ or GFP^−^, in bone marrows from recipients at 120 d (N = 3) post-transplantation were determined by FACS analysis. Data are represented as the mean ± SD. HSC-containing LSK cells and CD34^−^ LSK LT-HSC derived from Selp^−/−^ exhibited a competitive advantage. (C) The HSCs of *Selp* ^−*/*−^ self-renew significantly more than WT HSCs in 2nd transplantation experiment. The bone marrow cells from primary recipient were collected and transplanted into secondary recipients at 30 d post primary transplantation. The percentage of LSK cells and CD34^−^ LSK LT-HSC cells in the bone marrow of the recipients (*Selp* ^−*/*−^) cells were determined at 120 d post 2nd transplantation. Data are represented as the mean ± SD (N = 4). (D) Homing assay reveals decreased homing velocity for *Selp* ^−*/*−^ cells relative to UBC-GFP controls. Bone marrow cells from *Selp* ^−*/*−^ or WT mice (both CD45.2 background) were mixed in a 1∶1 ratio with UBC-GFP cells (CD45.2) and injected into CD45.1 recipients. Three hours post-injection, recipients were sacrificed and the percentages of CD45.2/GFP^+^ and CD45.2/GFP- cells were measured by FACS (representative FACS, left panel). The average ratios of WT to UBC-GFP or *Selp* ^−*/*−^ to UBC-GFP cells are presented. *Selp* ^−*/*−^ cells exhibited a significant homing defect relative to controls. (E) HSC homing assay reveals a decreased homing velocity for *Selp* ^−*/*−^ LSK cells relative to WT controls. Bone marrow cells from *Selp* ^−*/*−^ or WT mice were respectively injected into CD45.1 recipients (HSCs were normalized by FACS before injection). Three hours post-injection, recipients were sacrificed and the percentages of CD45.2^+^ LSK were measured by FACS (representative FACS, left panel). The average cell number (LSK) of WT or *Selp* ^−*/*−^ are presented. (F) Cell cycle analysis of WT and *Selp* ^−*/*−^ HSCs. Bone marrow cells were isolated from *Selp* ^−*/*−^ or WT mice. LSK cells were stained with Hoechst blue, and LSK cells in S+G2M phase of the cell cycle were examined by FACS. No significant differences were observed. For each experiment, the following denote the statistical significance observed: 0.01<P<0.05 =  *; 0.001<P<0.01 =  **; P<0.001 = ***.

We sought to characterize the underlying mechanism that enabled *Selp* ^−*/*−^ bone marrow cells to more efficiently repopulate the bone marrow space than WT cells. Using a competitive repopulation assay to test long-term engraftment (Figure 2A), HSC-containing LSK cells derived from *Selp* ^−*/*−^ mice (CD45.2^+^GFP^−^) and UBC-GFP mice (CD45.2^+^GFP^+^) were mixed in 1∶1 ratio and transplanted into irradiated CD45.1 recipients. Cells derived from *Selp* ^−*/*−^ recipients exhibited a competitive advantage over the cells derived from UBC-GFP mice by 45 d post-transplant that increased by 86 d and 120 d post-transplant (Fig. 2A). Recipients were sacrificed at 120 d post transplantation and characterized for the percentages of LSK cells and, in particular, LT-HSC (CD34^−^ LSK) cells in the bone marrow of the recipients (Fig. 2B). We noted that *Selp* ^−*/*−^ cells more efficiently engrafted in the bone marrow than WT cells, as indicated by increased levels of HSC-containing LSK cells and LT-HSCs (CD34^-^ LSK). We performed a secondary transplantation assay to characterize *Selp* ^−*/*−^ LSK self-renewal function (Fig. 2C). Primary CD45.1 recipients were transplanted with bone marrow cells from WT or *Selp* ^−*/*−^ mice. At 30 d, primary recipients were sacrificed and CD45.1 bone marrow cells were collected. Cells were normalized to ensure equivalent input of HSC-containing LSK cells prior to secondary transplantation into CD45.1 recipients. At 120 d post-secondary transplantation, a significantly higher percentage of *Selp* ^−*/*−^ HSC-containing LSK cells and LT-HSCs (CD34^−^ LSK) were detected in the bone marrow of recipients.

We tested the idea that the increased repopulation we observed was due to an enhanced homing velocity in *Selp* ^−*/*−^ cells by performing a competitive bone marrow homing assay (Fig. 2D). Bone marrow cells from the UBC-GFP mouse and those from *Selp* ^−*/*−^ mouse were mixed in a 1:1 ratio and then injected into CD45.1 mice. Percentages of CD45.2^+^GFP^+^ (WT) and CD45.2^+^GFP^−^ (*Selp* ^−*/*−^) cells in the bone marrow of CD45.1 recipient mice were compared after 3 h. These findings indicated that *Selp* ^−*/*−^ donor cells were not as efficient as WT cells in homing and that the enhanced repopulation of HSC-containing LSK cells observed in recipients of *Selp* ^−*/*−^ donor cells was not caused by an accelerated homing velocity. We then performed a HSC homing assay (Fig. 2E). LSK cells from *Selp* ^−*/*−^ or WT mice were normalized and injected into recipient mice. At 3 h post-transplant, animals were sacrificed and the numbers of HSC-containing LSK cells were counted. *Selp* ^−*/*−^ recipients possessed modestly lower levels of LSK cells than WT recipients. Although these data are not statistically significant, the results are consistent with the competitive bone marrow homing assay, which suggest a decreased homing velocity.

We next wanted to determine whether *Selp* ^−*/*−^ mice exhibited changes in HSC quiescence (Fig. 2F). Bone marrow cells isolated from *Selp* ^−*/*−^ and WT mice exhibited no changes in HSC quiescence, as measured by the percentage of LSK cells in the S + G2/M stages of the cell cycle.

### P-selectin expression exhibits an age-independent increase in expression on LT-HSCs

**Figure 3:**
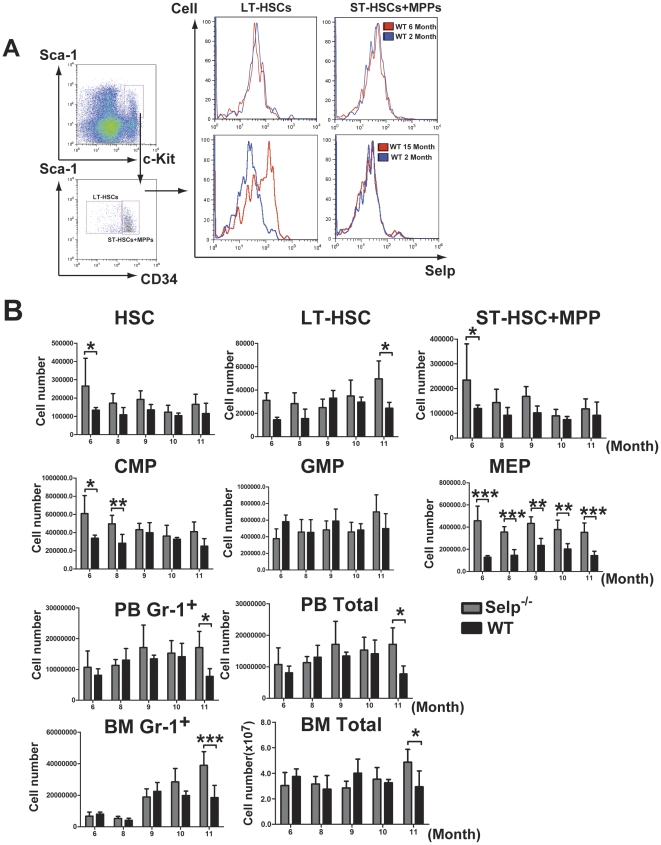
P-selectin expression on LT-HSCs increases with age but does not significantly alter HSC or progenitor populations. (A) LT-HSCs (CD34^−^ LSK) and ST-HSCs+MPPs (CD34^+^ LSK) were sorted by FACS from the bone marrow of 2 month-old, 6 month-old, and 15 month-old WT mice and tested for surface P-selectin expression (N = 3). Representative FACS show that CD34^−^ LSK LT-HSCs exhibit age-dependent increases in surface P-selectin expression but CD34^+^ LSK ST-HSCs + MPPs do not. (B) Data collected from FACS analyses of bone marrow cells (B) WT and *Selp* ^−*/*−^ mice aged 6, 8, 9, 10, or 11 months. Data represent the mean absolute numbers (± SD) of LSK, CD34^−^ LSK LT-HSC, CD34^+^ LSK ST-HSC + MPP, CMP, GMP, or MEP. Following two-way ANOVA, Bonferroni post-tests comparing *Selp* ^−*/*−^ mice to WT mice at each age point were executed. Significant differences between *Selp* ^−*/*−^ and WT HSC and progenitor populations were noted (0.01<P<0.05 =  *; 0.001<P<0.01 =  **; P<0.001 = ***).

P-selectin expression increases on the surface of HSCs with age [1]. We sorted LT-HSCs (CD34^−^ LSK) and ST-HSCs + MPPs (CD34^+^ LSK) from the bone marrow of 2 month-old, 6 month-old, and 15 month-old WT mice to determine whether the increase was restricted to either HSC subpopulation. No changes were noted between 2 month-old and 6 month-old mice; however, P-selectin expression on the surface of LT-HSCs was increased in 15 month-old mice. Expression on ST-HSCs + MPPs remained unchanged (Fig. 3A). These findings correlated with previous microarray and qRT-PCR data [2].

### Loss of P-selectin expression alters stem, progenitor, and mature cell fates in an age-dependent manner

*Selp* ^−*/*−^ and WT mice were aged for 6, 8, 9, 10, and 11 months. We collected bone marrow cells from these mice at each of these time points for FACS analysis of HSC-containing LSK cells and progenitor populations (Fig. 3B). We observed similar total numbers of bone marrow cells at 6, 8, 9, and 10 months of age, while there was a higher total of bone marrow cells in *Selp* ^−*/*−^ mice at 11 months of age when compared to the WT control. Overall, loss of *Selp* expression appeared to increase the total numbers of HSC-containing LSK cells in mice at 6 months of age, but this effect diminished with age. This finding corresponded with the levels of ST-HSC + MPPs that were observed. In contrast, there were increased levels of LT-HSCs (CD34^−^ LSK) in the *Selp* ^−*/*−^ mice when compared to controls at most time points, except at 9 months. At 11 months, there was a significant increase in the levels of LT-HSCs present. There were significantly more CMPs in *Selp* ^−*/*−^ than in WT mice at 6 and 8 months, but this difference dissipated by 9 months. While it had no effect on GMP populations, loss of *Selp* expression caused a marked expansion the MEP populations at each time point tested. We observed an increasing number of Gr1^+^ cells present in the bone marrow, with a significant difference noted at 11 months. This corresponded to Gr1^+^ levels that were observed in the peripheral blood. While no significant differences were observed in the numbers of total leukocytes present in the peripheral blood until 11 months, there was a marked increase in the Gr1^+^ cells in the peripheral blood by 9 months that remained through 10 and 11 months. These findings were indicative of strain- and age-dependent effects on stem, progenitor, and mature myeloid cell populations.

### Loss of *Selp* enhances the function of leukemia stem cells

**Figure 4:**
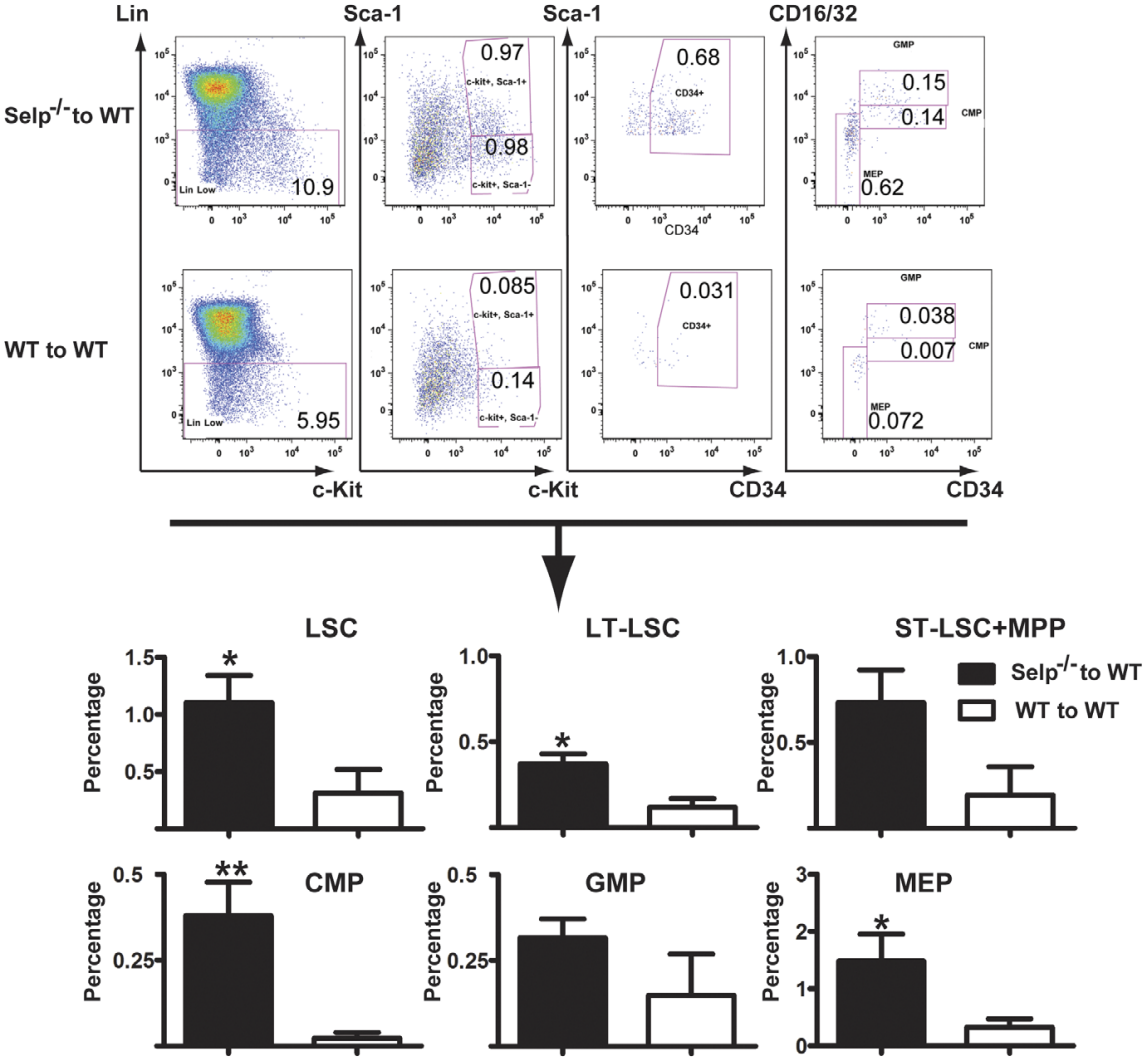
Recipients of 5-FU pretreated *Selp* ^−*/*−^ donor cells transduced to express BCR-ABL more efficiently repopulate the bone marrow of recipients than controls. (A) CML was induced in recipient mice using BCR-ABL-transduced bone marrow taken from 5-FU pre-treated donor *Selp* ^−*/*−^ or WT mice pre-stimulated with IL3, IL6, and SCF. Representative FACS (top panel) compares recipients of *Selp* ^−*/*−^ -derived cells to WT-derived cells. The mean data (bottom panel) revealed statistically significant elevations in the percentages of LSCs (CD34^−^ LSK LT-LSCs and CD34^+^ LSK ST-LSCs + MPPs) and progenitors (CMP, GMP, and MEP). (0.01<P<0.05 =  *; 0.001<P<0.01 =  **)

Because loss of *Selp* expression enhances HSC function (Figure 1,2), we examined whether *Selp* also regulates the function of leukemia stem cells (LSCs) using a BCR-ABL-induced CML as a disease model. CML is a stem cell disease, and we have previously identified LSCs for CML in mice [10]. We have previously shown that loss of *Selp* causes acceleration of CML development [4], but it is unknown whether this is caused by a P-selectin-mediated effect resulting from its expression on the surface of LSCs. Bone marrow cells from 5-FU-treated donor WT and *Selp* ^−*/*−^ mice were infected with retrovirus expressing BCR-ABL-GFP. Irradiated WT recipients were transplanted with 2×10^5^ of these transduced donor cells. At 14 d post-transplant, recipient mice from each of the groups were sacrificed, and bone marrow cells were harvested and analyzed by FACS (Fig. 4). Recipients of leukemic *Selp* ^−*/*−^ cells possessed significantly more LSC-containing LSK cells than recipients of WT cells. There were more LT-LSCs (CD34^-^ LSK) and ST-LSCs + MPPs (CD34^+^ LSK) in recipients of *Selp* ^−*/*−^ cells than WT cells. In addition, recipients of leukemic *Selp* ^−*/*−^ cells possessed significantly more CMP and MEP cells.

### Mouse recipients of *Selp* ^−*/*−^ BCR-ABL-expressing donor cells develop a more insidious CML-like disease

**Figure 5:**
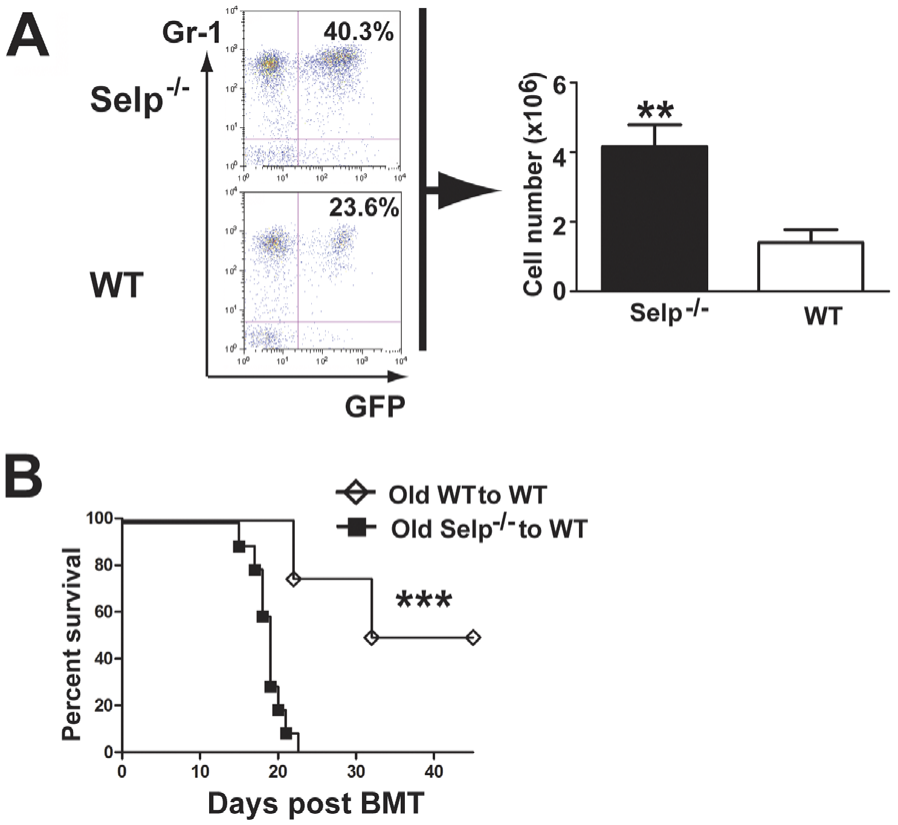
Recipients of *Selp* ^−*/*−^ derived from 15 month old donors develop a more aggressive CML-like disease than age-matched controls. CML was induced in recipient mice using BCR-ABL-transduced bone marrow cells taken from 5-FU treated 15 month old donor *Selp* ^−*/*−^ or WT mice pre-stimulated with IL3, IL6, and SCF. FACS analysis (A, left panel) showed GFP^+^Gr-1^+^ cells in peripheral blood of recipients of BCR-ABL transduced *Selp* ^−*/*−^ and WT cells from old donors. (A, right panel) At 13 d post-transplant, *Selp* ^−*/*−^ recipients (N = 10) possessed significantly more GFP^+^Gr1^+^ cells in the peripheral circulation than WT recipients (N = 9). (B) Kaplan-Meier survival analysis revealed an accelerated form of CML in recipients of aged *Selp* ^−*/*−^ marrow cells (N = 10) retrovirally transduced with BCR-ABL when compared to recipients of WT bone marrow cells (N = 9) retrovirally transduced with BCR-ABL. For each experiment, the following denote the statistical significance observed: 0.01<P<0.05 =  *; 0.001<P<0.01 =  **; P<0.001.

Bone marrow cells derived from 15-month-old donor *Selp* ^−*/*−^ and WT mice were transduced with BCR-ABL, respectively, followed by transplantation of the transduced cells into recipient mice. Leukemia cells (GFP^+^/Gr1^+^) in peripheral blood of recipients of BCR-ABL transduced *Selp* ^−*/*−^ cells are significant higher than those in recipients of the transduced WT cells (Figure 5A). All recipients of BCR-ABL transduced *Selp* ^−*/*−^ cells died by 23 d after induction of CML and had a median survival of 19 d, whereas recipients of the transduced WT cells survived significantly longer (Fig. 5B).

## Discussion

In a previous study, we showed that *Selp* ^−*/*−^ recipients of *Selp* ^−*/*−^donor bone marrow transduced by BCR-ABL developed a more aggressive form of CML-like disease than the recipients of WT cells [4]. We demonstrated that in the absence of P-selectin expression in both donor cells and recipient stroma, BCR-ABL-expressing progenitors are prematurely released from the bone marrow due to an apparent adhesion defect; and this leads to significant pulmonary hemorrhages and cellular masses due to massive infiltration of myeloid leukemia cells. P-selectin expression has been associated with platelets and endothelial cells [3] and is known to contribute to variety of processes including coagulation, inflammation, and tumor metastasis [11], [12]. The traditional view was that stromal, P-selectin-expressing endothelial cells interact with P-selectin ligand-bearing (PSGL1) leukocytes and facilitate an assortment of processes including adhesion and rolling and cellular activation [13]. In addition, platelets expressing P-selectin are capable of binding directly to leukocytes, contributing to rolling, and to tumor cells, supporting metastasis [12]. Recent findings that HSCs express P-selectin [14] and the finding that the level of P-selectin expression on LT-HSCs increases with age (Ref. [2] and Figure 3A) confound the current understanding of its role in mediating the leukocyte-stroma interaction [1], [14] and may have important implications for leukemogenesis [2]. This study addressed questions related to how surface expression of P-selectin affects HSC and LSC populations and function. Indeed, we have demonstrated that P-selectin expression on the surface of HSCs and progenitors is critical to their function and thus normal myelopoiesis and may contribute to Ph^+^ chronic myeloid leukemogenesis. We showed that the loss of P-selectin expression facilitated bone marrow repopulation by LSK populations including LT-HSCs (CD34^−^) and ST-HSCs + MPPs (CD34^+^) (Fig. 1). In addition, significant differences in progenitor populations were noted. These differences were cell-dependent, as *Selp* ^−*/*−^ cells were not more susceptible to viral transduction than WT cells. Furthermore, we cultured of *Selp* ^−*/*−^ and WT LSK cells *in vitro* and noted no significant differences in growth kinetics after 4 d of culture. This capacity was also observed at the HSC level, where *Selp* ^−*/*−^ LSK cells exhibited a long-term competitive advantage over WT LSK cells (Figure 2B). This competitive advantage at the stem cell level led to the increased number of *Selp* ^−*/*−^ cells present in the peripheral circulation (Fig. 2A), as well as in the bone marrow space (Fig. 2B). We attributed the advantage *Selp* ^−*/*−^ cells possess over WT cells to an enhanced capacity for self-renewal (Fig. 2C). We observed that this functional advantage was not attributable to an enhanced homing velocity, as WT cells homed more efficiently than *Selp* ^−*/*−^ (Figure 2D–E). These findings are surprising in that we had previously shown through an alternative method that *Selp* ^−*/*−^ and WT cells homed with similar efficiencies when introduced into recipients of the same genetic background [4]. In this paper, the approach differed and relied upon a CD45.1 recipient. Thus, effects from different microenvironments (*Selp* ^−*/*−^ vs. WT) on donor cell homing could be avoided. This enabled us to focus our homing assay on the donor cells and not the recipient stroma. Our findings indicate an initial competitive disadvantage for *Selp* ^−*/*−^ donor cells because of a presumed inability to be retained in the bone marrow space. We speculate that the premature release of *Selp* ^−*/*−^ cells from the bone marrow may result in a compensatory increase in levels of *Selp* ^−*/*−^ cells. Alternatively, it is possible that other adhesion mechanisms may predominate in spite of *Selp* expression or at least may compensate for loss of function when *Selp* is not expressed. In the long term, *Selp* ^−*/*−^ cells possess a competitive advantage due to enhanced stem cell self-renewal properties.

We have previously shown that BCR-ABL-transformed HSCs exhibiting the LSK expression profile function as *de facto* CML stem cells [10]. In this paper, we demonstrate that in the absence of P-selectin expression on LSCs, a more aggressive form of CML develops, as indicated by the elevated percentage of LSCs observed in the bone marrow (Fig. 4) and the decline in mean survival time following transplantation of leukemic cells (Fig. 5). The aggression with which CML proceeds is dependent on the donor LSC/progenitor. In fact, both LT-LSC (CD34^−^ LSK) and ST-LSC + MPP (CD34^+^ LSK) percentages were increased in the bone marrow, and, as a result, the total number of leukemic cells in peripheral blood was increased. The increase in the percentage of LSCs is mostly attributable to the cell-intrinsic differences related to the expression of P-selectin. It has been speculated that LT-HSCs, when transformed, may actually be the true stem cell source for most myeloid leukemias [2]. With aging, there is an increase in LT-HSCs (Fig. 3); however, unlike LT-HSCs found in younger people, aged LT-HSC are often homeostatically disrupted [15], and exhibit increasing levels of self-renewal and an unusual propensity to generate more myeloid than lymphoid progenitors [2]. This disruption in the lymphoid/myeloid balance, coupled with the overall elevation in LT-HSCs, contributes to making Ph^+^ CML more common with age (according to NCI SEER 2001–2005, median age of diagnosis in the U.S.  = 66 yrs. old; >50% of incidences diagnosed ≥65 yrs. old). We have shown in HSC-containing LSK cells that loss of *Selp* expression increased the capacity for their self-renewal (Fig. 2). In the context of CML, we postulate that the loss of *Selp* expression increases LSC self-renewal, thereby accelerating leukemogenesis. In older mice, loss of *Selp* expression may have additive or synergistic effects on leukemia development.
